# Effects of Unilateral High Frequency Stimulation of the Subthalamic Nucleus on Risk-avoidant Behavior in a Partial 6-hydroxydopamine Model of Parkinson’s Disease

**DOI:** 10.31083/j.jin2304084

**Published:** 2024-04-19

**Authors:** Sydney G. Hillan, Anders J. Asp, Leena B. Pramanik, Aarushi A. Mukerjee, Carter B. Mulder, Wendy D. Lujan, Jodi L. Silvernail, Su-Youne Chang, Suelen L. Boschen, J. Luis Lujan

**Affiliations:** 1 Mayo Clinic Graduate School of Biomedical Sciences, Mayo Clinic, Rochester, MN 55905, USA; 2 Department of Physical Medicine and Rehabilitation, Mayo Clinic, Rochester, MN 55905, USA; 3 Department of Neurologic Surgery, Mayo Clinic, Jacksonville, FL 32224, USA; 4 Department of Psychiatry and Behavioral Sciences, University of Minnesota, Minneapolis, MN 55454, USA; 5 Department of Neurologic Surgery, Mayo Clinic, Rochester, MN 55905, USA; 6 Department of Physiology and Biomedical Engineering, Mayo Clinic, Rochester, MN 55905, USA

**Keywords:** deep brain stimulation, subthalamic nucleus, risk-avoidance, 6-hydroxydopamine, Parkinson’s disease

## Abstract

**Background:**

Deep brain stimulation (DBS) of the subthalamic nucleus (STN) is a well-established treatment for the motor symptoms of Parkinson’s disease (PD). While PD is primarily characterized by motor symptoms such as tremor, rigidity, and bradykinesia, it also involves a range of non-motor symptoms, and anxiety is one of the most common. The relationship between PD and anxiety is complex and can be a result of both pathological neural changes and the psychological and emotional impacts of living with a chronic progressive condition. Managing anxiety in PD is critical for improving the patients’ quality of life. However, patients undergoing STN DBS can occasionally experience increased anxiety.

**Methods:**

This study investigates changes in risk-avoidant behavior following STN DBS in a pre-motor animal model of PD under chronic and acute unilateral high frequency stimulation.

**Results:**

No significant changes in risk-avoidant behaviors were observed in rats who underwent STN DBS compared with sham stimulation controls. Chronic stimulation prevented sensitization in the elevated zero maze.

**Conclusions:**

These results suggest that unilateral stimulation of the STN may have minimal effects on risk-avoidant behaviors in PD. However, additional research is required to fully understand the mechanisms responsible for changes in anxiety during STN DBS for PD.

## 1. Introduction

Deep brain stimulation (DBS) is an effective treatment for reducing the motor symptoms of Parkinson’s disease (PD) [1,2]. The primary stimulation target for PD is the subthalamic nucleus (STN), a brain region within the basal ganglia, a complex network involved in motor control, cognition, and emotional regulation [3]. The STN plays a critical role in motor control and is associated with the regulation of movement through its connections with other parts of the basal ganglia circuitry [4]. However, the STN also has connections with associative regions of the brain involved in cognitive function and limbic regions associated with emotional functions [5].

PD is a neurodegenerative disorder characterized primarily by motor symptoms such as tremor, rigidity, and bradykinesia [6]. However, PD also involves a wide range of non-motor symptoms [7] such as anxiety. Although anxiety occurs in up to 52% of PD patients [8], the impact of STN DBS on anxiety has yet to be characterized [9]. Some studies have reported an improvement in anxiety [10–15] while others have reported a worsening of anxiety following STN DBS [14,16–18]. There is a higher prevalence of PD in males compared with females [19]. As such, a large percentage of the population undergoing STN DBS are male [20]. However, anxiety is more prevalent in females [21]. In fact, previous studies have shown greater improvement in psychiatric symptoms following STN DBS in males compared with females [20]. Thus, the underlying mechanisms responsible for these distinct anxiogenic and anxiolytic effects remain elusive and could be the result of a myriad of factors such as differences in neuroanatomy, neuronal degeneration, pharmacological interventions, comorbidities, sex, and other factors unique to each patient.

Shared neural circuitry such as the amygdala, prefrontal cortex, and the hypothalamic-pituitary-adrenal (HPA) axis in certain animal models has been found to parallel those in humans, thus enabling investigation of anxiety-related processes [22,23]. Additionally, pharmacological interventions have been found to produce similar anxiolytic and anxiogenic effects in both humans and these animal models [24,25]. Furthermore, anxiety in both humans and rats is characterized by risk-avoidant behaviors, increased vigilance, and fear responses [26–31]. These commonalities in neurobiology, behavior, and pharmacological responses reinforce the validity of such models for studying pathological mechanisms and evaluating therapeutic interventions. In 2013, Creed *et al*. [32] showed that neither STN nor entopeduncular stimulation caused a significant change in risk-avoidant behaviors in naïve rats. Subsequently, Faggiani *et al*. [33] demonstrated an increase in risk-avoidant behaviors in multiple monoamine depletion models but a decrease in risk-avoidant behaviors following acute bilateral STN stimulation. Expanding on this, Badstuebner *et al*. [34] showed a decrease in risk-avoidant behaviors in a hemiparkinsonian model following long-term unilateral STN stimulation in an open field test. While these studies have investigated the effect of STN DBS on risk-avoidant behaviors, there is a need to better understand the impact of varying modes of stimulation on such behaviors.

The relationship between PD and anxiety is complex and can be a result of both pathological changes in neuronal activity or other psychological and emotional factors. This study builds on the current literature by investigating the effect of chronic and acute unilateral STN stimulation on risk-avoidant behaviors in a premotor model of PD. Elucidating these effects could lead to an improved understanding of the underlying pathophysiological mechanisms of PD and its motor and non-motor symptoms, in addition to furthering our knowledge of the effects of STN DBS.

## 2. Materials and Methods

### 2.1 Animal Model and Experimental Groups

In this study, we investigated changes in risk-avoidant behaviors. Adult male (n = 37) and female (n = 35) Sprague Dawley rats (Envigo, Indianapolis, IN, USA) weighing 200–400 g underwent a partial bilateral 6-hydroxydopamine (6-OHDA)-induced dopaminergic lesion to model the premotor stages of PD [35,36]. The estrous cycle of the female rats was not considered in the analysis. Traditional fully lesioned hemi-parkinsonian 6-OHDA models show decreased rodent mobility [37], while the partially lesioned premotor model used in this study allows for the non-motor symptoms of PD to emerge prior to the presentation of the motor symptoms. Clinical DBS is not typically performed at this stage of neural degeneration in PD. However, the selected lesion severity is more consistent with the time when the non-motor symptoms of PD start to manifest [38]. Furthermore, this model reduces confounds from mobility restrictions ensuing from full lesions [35,36] during assessment of risk-avoidant behaviors. The dopaminergic lesion level was confirmed using tyrosine hydroxylase (TH) immunohistologic analysis (see Immunohistologic Analysis section below). Rats were housed in a social environment prior to surgery in accordance with the Institutional Animal Care and Use Committee (IACUC) standards. Rats had ad libitum access to food and water and were kept on a 12-hour light/dark cycle. All behavioral tests were performed during the light portion of the light/dark cycle. All rats were housed individually following surgery.

Rats were divided into four experimental groups: lesioned undergoing chronic stimulation (n = 10), lesioned undergoing acute stimulation (n = 18), lesioned undergoing sham stimulation (n = 12), and vehicle controls (n = 10; no lesion and no DBS electrode). An additional 22 rats were used for anxiogenic caffeine control experiments, with 12 rats used for the elevated zero maze test, which received 100 mg of caffeine, and 10 rats used for the open field test, which received 50 mg of caffeine.

### 2.2 Surgical Procedures: Dopaminergic Lesion and Electrode Implantation

Anesthesia was induced using 3% isoflurane (NDC 66794-019-10, Piramal Critical Care, Inc, Bethlehem, PA, USA) and maintained with 1%–2% isoflurane throughout the procedure. Rats received 1 mg/kg buprenorphine (NDC 79926-058-17, Wedgewood Connect, San Jose, CA, USA) subcutaneously for analgesia prior to surgery. Burr holes were drilled above the striatum (Medial Lateral, ML ± 2.7, Anterior Posterior, AP +1.2, Dorsal Ventral, DV −4.2) [39] and STN (ML 2.5, AP −3.6, DV −7.8) [39] regions. To induce the dopaminergic lesion, 5 μg 6-OHDA (Sigma-Aldrich, Milwaukee, WI, USA) was first dissolved in a 2.5 μL saline solution containing 0.02% ascorbic acid (Sigma-Aldrich) and bilaterally injected into the striatum at a rate of 500 nL/min using a Hamilton syringe (Hamilton Company, Reno, NV, USA) and micro syringe pump (UMP3 Ultramicropump, World Precision Instruments, Sarasota, FL, USA). Rats in the non-lesioned group (vehicle controls) received a saline solution containing 0.02% ascorbic acid. All rats received a subcutaneous injection of 25 mg/kg desipramine (Sigma-Aldrich) 15–30 minutes prior to surgery to prevent noradrenergic neuron degeneration. A bipolar platinum-iridium electrode (MS303/8C, Plastics One, Roanoke, VA, USA) was implanted unilaterally in all groups into the right STN. The electrode was secured in place using four skull screws (MD-1310, BASi, West Lafayette, IN, USA) covered with a Metabond (Parkell, Edgewood, NY, USA) headcap and fixed with dental cement. Rats were allowed to recover for 2 weeks prior to electrical stimulation and behavioral testing.

### 2.3 Electrical Stimulation

**Fig. 1.**
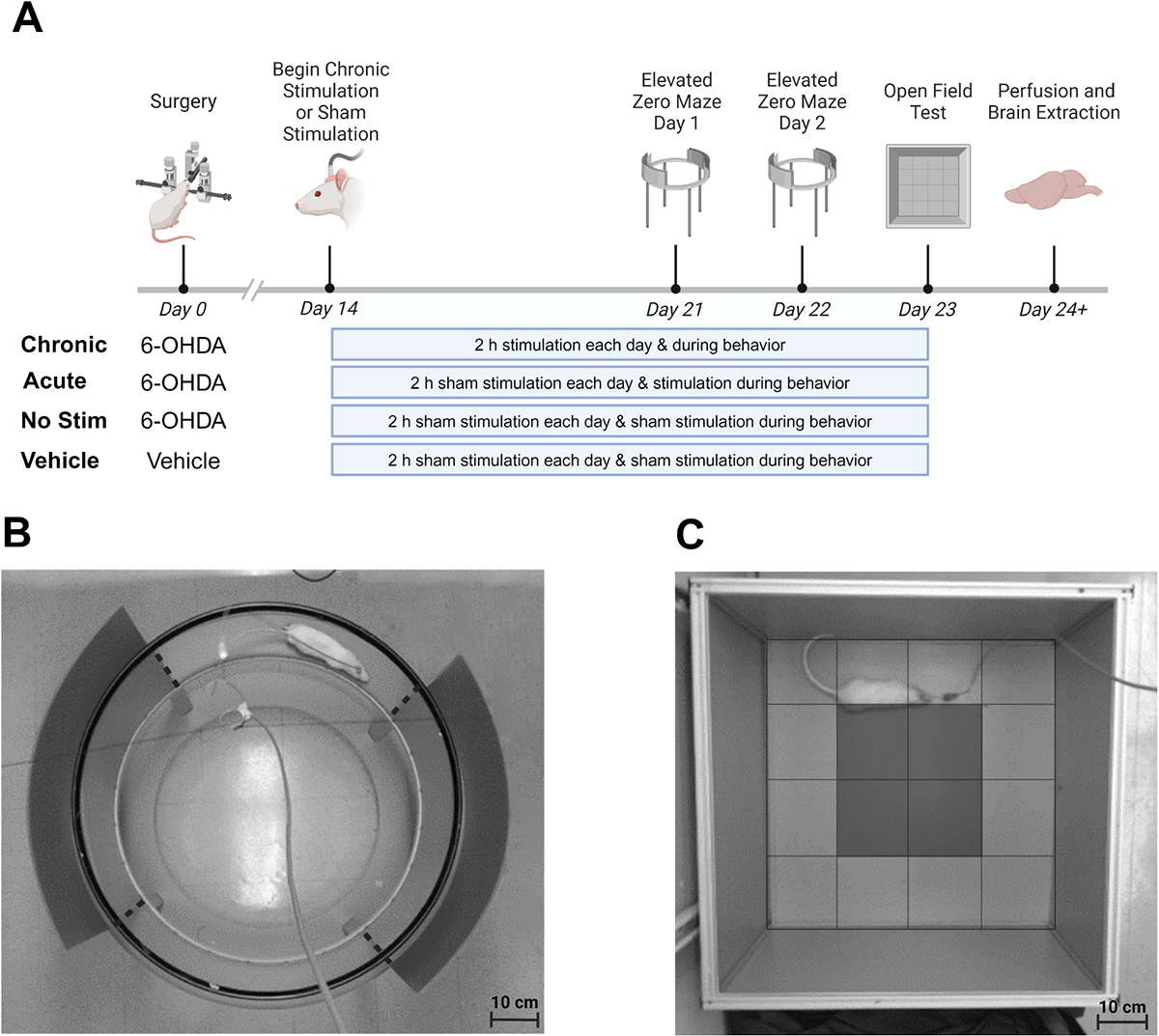
Experimental approach. (A) Experimental timeline and test groups. Surgery to induce dopaminergic 6-hydroxydopamine (6-OHDA) lesioning or vehicle injection and deep brain stimulation electrode implantation was performed on day 0. Sham and chronic stimulation (when appropriate) began on day 14 and continued until day 23. Behavioral analysis using the elevated zero maze was performed on day 21 and again on day 22. The open field test was performed on day 23. Perfusion was performed following completion of all behavioral tests. (B) Elevated zero maze setup. Rats that spent more time in the closed arms exhibited behavior associated with anxiety and fear, while rats that spent more time in the open arms were considered less anxious and more willing to explore open environments. Dashed lines indicate the areas where the closed and open arms intersect and are used to quantify exploratory behavior. (C) Open field test setup. The shaded region indicates the center of the field used to quantify exploratory behavior. Anxious rats spent less time in the central, shaded, and more exposed areas of the field. The open field test was also used to quantify total distance traveled as a measure of locomotor function.

Rats underwent unilateral STN DBS at 130 Hz with 60-μs charge-balanced biphasic pulses delivered using a stimulator built in-house [40]. Stimulation amplitude was determined for each animal by first identifying the minimum amplitude that evoked dyskinetic activity (motor threshold) [41]. Rats were stimulated at 80% of their motor threshold, which ranged from 50 to 300 μA. Rats who did not exhibit dyskinetic activity when stimulated at 300 μA were considered non-responders and excluded from the study, as stimulating above that threshold could cause significant tissue damage [42]. All rats were tethered to the stimulator for 2 hours a day starting on day 14 and continuing for the remainder of the study (day 23). Rats in the chronic stimulation group underwent daily stimulation for 2 hours starting on day 14 and continuing for the remainder of the study (day 23) (Fig. 1A). Acute rats were only stimulated during the behavior tests on days 21 to 23. Rats were kept in their home cages during this period of chronic stimulation or tethering.

### 2.4 Behavioral Analyses

Elevated Zero Maze: an elevated zero maze was used to assess anxiety-related behaviors (time spent in open and closed arms, number of entries into closed arms, and number of head dips) in rats. The elevated zero maze consisted of a circular, 100 cm in diameter elevated platform with 10-cm wide tracks with two enclosed quadrants (closed arms) providing a sense of safety and security and two open quadrants (open arms) exposing the rat to the open, elevated environment (Fig. 1B). Prior to their placement in the elevated zero maze, rats were tethered and stimulated (when appropriate) in their home cage for 2 hours prior to testing. Habituation to the room with the elevated zero maze took place for 10 minutes while the rats were in their home cages. Following habituation, rats were placed in the elevated zero maze and allowed to move freely while tethered to the electrical stimulator, which was suspended above the maze. The test was conducted over 2 days to investigate the impact of a second day of testing. The first day of the elevated zero maze test (day 21) was used to capture animal behavior in a new environment, while the second day (day 22) was used to capture animal behavior in a non-novel environment [30,43]. Rats were placed in the maze at one of the four intersections (Fig. 1B, dashed lines) between the open and closed arms but facing the closed arms. The specific intersection selected was varied for each animal. On day 21, rats were allowed to explore the maze for 10 minutes. On day 22, rats were allowed to explore the maze for 5 minutes. Times were determined based on the literature [43,44]. Only the first 5 minutes of day 21 were analyzed to account for the difference in time between days 21 and 22. Stimulation was delivered to the acute and chronic experimental groups during the entire time rats were connected to the stimulator. Cameras placed directly above the maze recorded animal behavior, which was analyzed using AnyMaze (Stoelting, Chicago, IL, USA). Time spent in the open and closed arms as well as total distance traveled were also measured using AnyMaze. Entries into the closed arm of the maze, defined as 75% of the animals entering the closed arm, were determined by video analysis. Head dips, defined as the times the animal’s head dropped below the bottom of the maze while in the elevated open arms, were determined by video analysis. Open Field Test: risk-avoidance and mobility were characterized using an open field test setup 60 cm in length, 60 cm in width, and 44 cm in height (Fig. 1C). A high-definition video camera and stimulator were placed directly above the field to allow for simultaneous stimulation and video recording while the rats moved freely in the field. Time spent in the center of the field (defined as 15 cm from the wall of the field) and the total distance traveled were measured using EthoVision (Noldus, Wageningen, the Netherlands). On the day of testing, rats were tethered and connected to the stimulator for 2 hours in their home cage, and this was followed by 10 minutes of habituation and 5 minutes of behavioral testing inside the open field setup. Stimulation was delivered to the acute and chronic experimental groups during the entire time rats were connected to the stimulator.

### 2.5 Anxiogenic Behavior Verification

Two additional experimental groups of rats received an intraperitoneal (IP) injection of either caffeine or saline to validate the ability to detect anxiogenic behaviors using the elevated zero maze and open field test. These rats received no lesion. Caffeine (Sigma Aldrich) was administered at 100 mg/kg for the elevated zero maze and 50 mg/kg for the open field test [44,45]. Rats were introduced to each test 30 minutes following administration of caffeine or saline. Testing was conducted following the same approach used for the stimulation experiments described previously.

### 2.6 Immunohistologic Analysis

Once all experiments were completed, rats were perfused by exposing the heart and major blood vessels under pentobarbital (Vortech Pharmaceuticals, Ltd., Dearborn, MI, USA)-anesthesia (100 mg/kg). Saline solution followed by 4% paraformaldehyde (PFA) solution (Sigma-Aldrich) were used to flush the circulatory system and replace the blood with the fixative solution prior to brain extraction. Circulatory flush was performed using an infusion pump (KD Scientific, Holliston, MA, USA) at a rate of 60 mL/min. The brain was fixed overnight in 4% PFA and then submerged in a 25% glycerol sinking solution for approximately 5 days to prepare tissue for sectioning and preserve cellular structures for further analysis. Extracted brains were frozen in dry ice and cut into 40-μm sections using a microtome (Leica, Nussloch, Germany). TH was used to determine the percentage of dopamine degeneration in the substantia nigra following the 6-OHDA lesion, quantified by optical density (ImageJ, Bethesda, MD, USA). Brain sections were rinsed in a phosphate-buffered saline (PBS) with 0.2% Triton X (Tx), soaked in 3% hydrogen peroxide for 10 minutes, and rinsed and blocked with a 10% natural goat serum in PBS blocking buffer (Vector Laboratories, Newark, CA, USA) for 1 hour at room temperature (23 °C). Brain sections were then incubated in anti-TH antibody (Rabbit) (Millipore Sigma, Darmstadt, Germany) diluted in the blocking buffer overnight at 4 °C. Following PBS-Tx rinsing, sections were incubated with Biotinylated goat anti rabbit Ig (Vector Laboratories) diluted in block (10% natural goat serum) for 1 hour, then rinsed in PBS-Tx and incubated for 1 hour in Avidin-biotin blocking reagent (VECTASTAIN ABC-HRP Kit, Vector Laboratory, Newark, CA, USA). Sections were rinsed in phosphate buffer and incubated in Vector DAB substrate (Vector Laboratory) for 5 minutes until sections had completed staining. Sections were mounted onto glass slides for imaging and brightfield images were collected using an AxioScan microscope (ZEISS, Oberkochen, Germany). Optical density was calculated by first identifying a region of interest around the substantia nigra and measuring the pixel intensity within the region. Next, background levels were subtracted from the pixel intensity values. Finally, optical density was calculated using ImageJ [46]. Electrode location was confirmed using cresyl violet (Sigma-Aldrich) staining.

### 2.7 Statistical Analysis

Data normality was assessed for all data sets using a Shapiro-Wilk test. Nonparametric statistical tests were chosen as the most appropriate approach due to the small sample size and non-normal distribution of the data. All statistical analyses were conducted using GraphPad Prism version 10.0.0 (GraphPad, Boston, MA, USA). For data sets involving more than two unpaired cohorts with a nonparametric distribution, the Kruskal-Wallis test was used to determine if there was a significant difference between the groups. If the Kruskal-Wallis test determined there were no significant differences between the groups (i.e., *p* > 0.05), then the Kruskal-Wallis statistic H and the *p* value of the Kruskal-Wallis test were calculated for the experiment. If the Kruskal-Wallis test identified a significant difference between the groups (i.e., *p* < 0.05), then Dunn’s multiple comparison test was used as a post-hoc test to determine which groups were significantly different from each other and the *p* value for the Dunn’s comparison was shown. The Mann-Whitney test was used to compare nonparametric data between unpaired cohorts, and the results were reported with the Mann-Whitney U statistic and the *p* value. The Wilcoxon matched pairs signed rank test was applied for paired cohorts, and the results shown with the sum of signed ranks (W) and the *p* value. Statistical significance was determined at *p* < 0.05. The median and interquartile range are presented on each graph. Outliers were identified using the ROUT method with a Q value of 1% (GraphPad, Boston, MA, USA) and removed from the analysis.

## 3. Results

### 3.1 Risk-Avoidant Behaviors: Elevated Zero Maze and Open Field Test

**Fig. 2.**
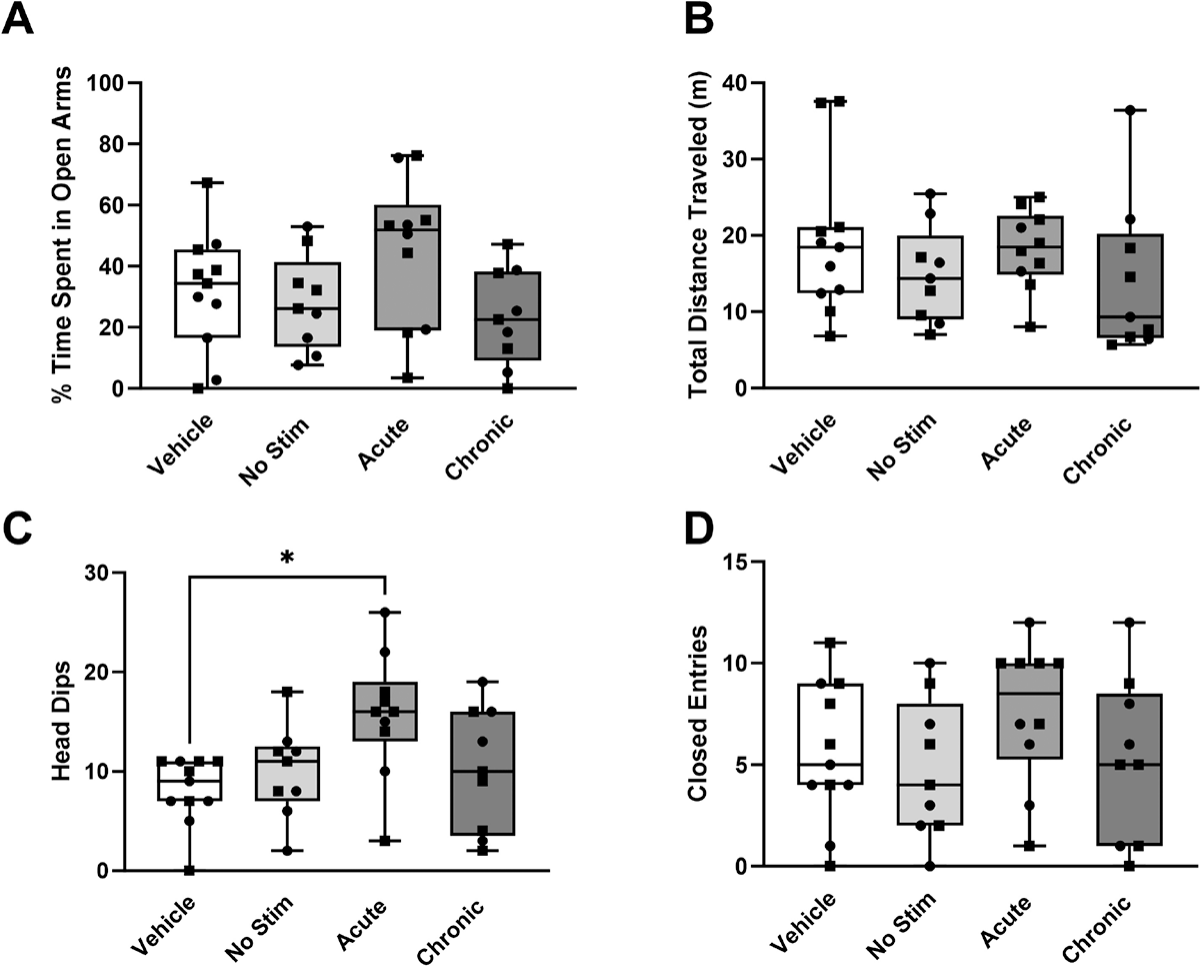
Risk-avoidant behavior in the elevated zero maze at day 21. Risk-avoidant behavior for the first 5 minutes that rats spent in the elevated zero maze for each of the experimental groups: chronically stimulated (n = 10), acutely stimulated (n = 10), lesioned but not stimulated (n = 9), and implanted but not-stimulated vehicle (not-lesioned) controls (n = 11). Subthalamic nucleus deep brain stimulation did not evoke significant changes in (A) the time spent in the open arms (H = 5.62, *p* = 0.132), (B) total distance traveled (H = 3.891, *p* = 0.273), and (D) entries into the closed arms (H = 3.80, *p* = 0.284) compared with rats who were not stimulated. However, a significant change was observed in (C) head dipping behavior between stimulated and non-stimulated groups *p* < 0.05 (H = 9.30, *p* = 0.026). Specifically, acute stimulation resulted in an increase in the number of head dips compared with the vehicle control group (*p* = 0.017). Data are presented using box and whisker plots. The median is denoted by the line inside the box, while the box edges represent the lower (25%) and upper (75%) quartiles. Squares represent female rats and circles represent male rats. A *p* value of 0.05 was considered significant using the Kruskal-Wallis test * *p* < 0.05).

We quantified the percentage of time spent in the open arms of the elevated zero maze for all experimental groups. The chronically stimulated group had a median time spent in the open arms of 22.57%, while the acutely stimulated group had a median time spent of 51.83% (Fig. 2A). No statistically significant difference was observed between any of the experimental groups (H = 5.62, *p* = 0.132). Additionally, there were no significant differences in the total distance traveled (Fig. 2B, H = 3.89, *p* = 0.273) or closed arm entries (Fig. 2D, H = 3.80, *p* = 0.284) between the experimental groups. However, the acutely stimulated group showed significantly more head dips (Fig. 2C, *p* = 0.017) compared with the vehicle group.

**Fig. 3.**
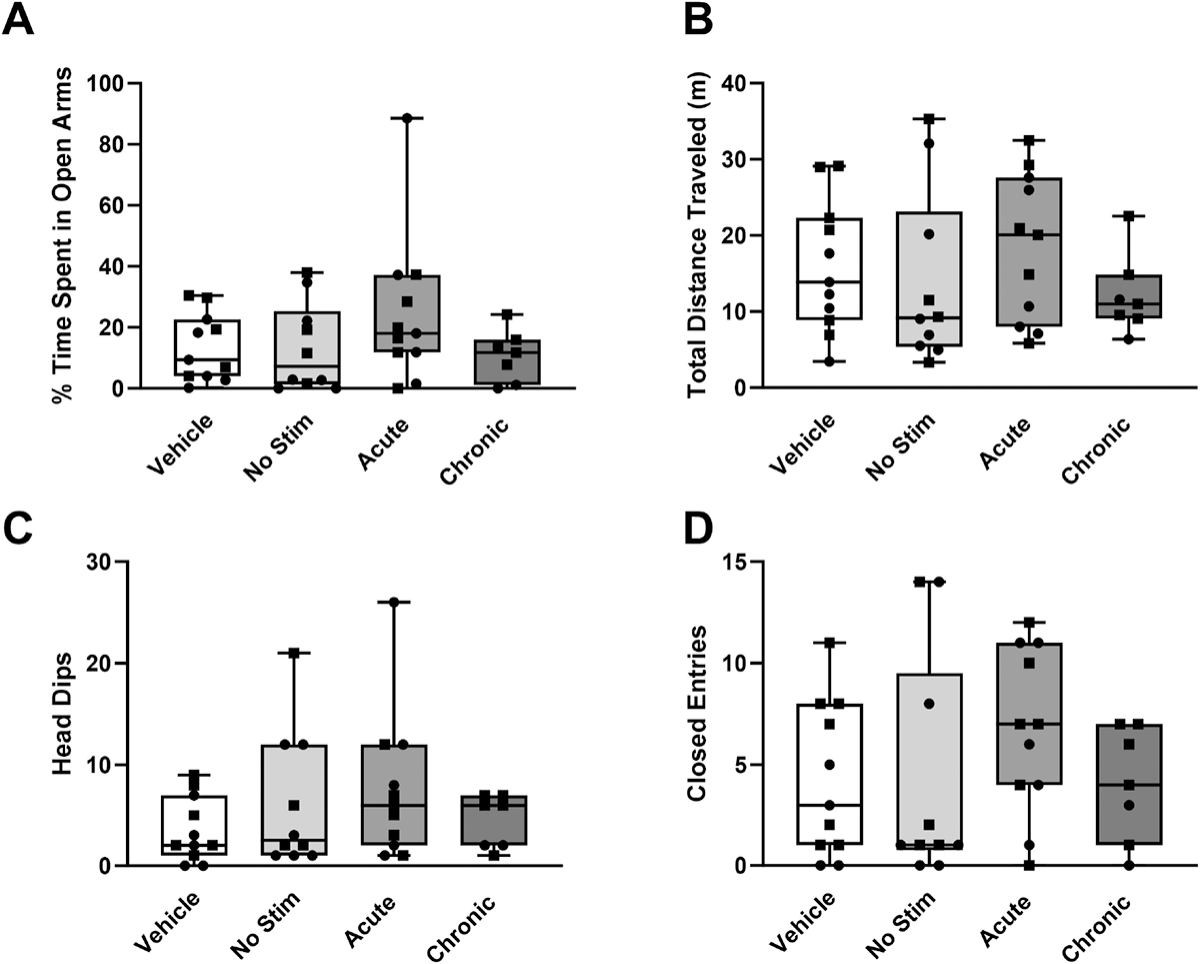
Risk-avoidant behavior in the elevated zero maze at day 22. Risk-avoidant behavior for the 5 minutes that rats spent in the elevated zero maze for each of the experimental groups: chronically stimulated (n = 7), acutely stimulated (n = 11), lesioned but not stimulated (n = 10), and implanted but not-stimulated vehicle (not-lesioned) controls (n = 11). Subthalamic nucleus deep brain stimulation did not evoke significant changes in (A) percentage of time spent in open arms (H = 2.58, *p* = 0.461), (B) total distanced traveled (H = 2.36, *p* = 0.501), (C) number of head dips (H = 2.15, *p* = 0.542), or (D) number of entries into the closed arms (H = 2.73, *p* = 0.435) compared with rats that were not stimulated. Data are presented using box and whisker plots. The median is denoted by the line inside the box, while the box edges represent the lower (25%) and upper (75%) quartiles. Squares represent female rats and circles represent male rats. A *p* value of 0.05 was considered significant using the Kruskal-Wallis test.

We conducted an additional behavioral assessment in the elevated zero maze on the subsequent day, and results were consistent with those observed in the initial exposure to the maze during the previous day. No statistically significant differences (H = 2.58, *p* = 0.461) in the percentage of time spent in the open arms were observed between experimental groups (Fig. 3A). However, a slight increase in the percentage of time spent in the open arms was observed in the acute group. No significant differences (H = 2.36, *p* = 0.501) were observed in the total distance traveled between experimental groups (Fig. 3B). Secondary measurements, including the number of head dips and closed arm entries did not result in statistically significant differences (H = 2.15, *p* = 0.542 and H = 2.73, *p* = 0.435, respectively) between the experimental groups (Fig. 3C,D).

**Fig. 4.**
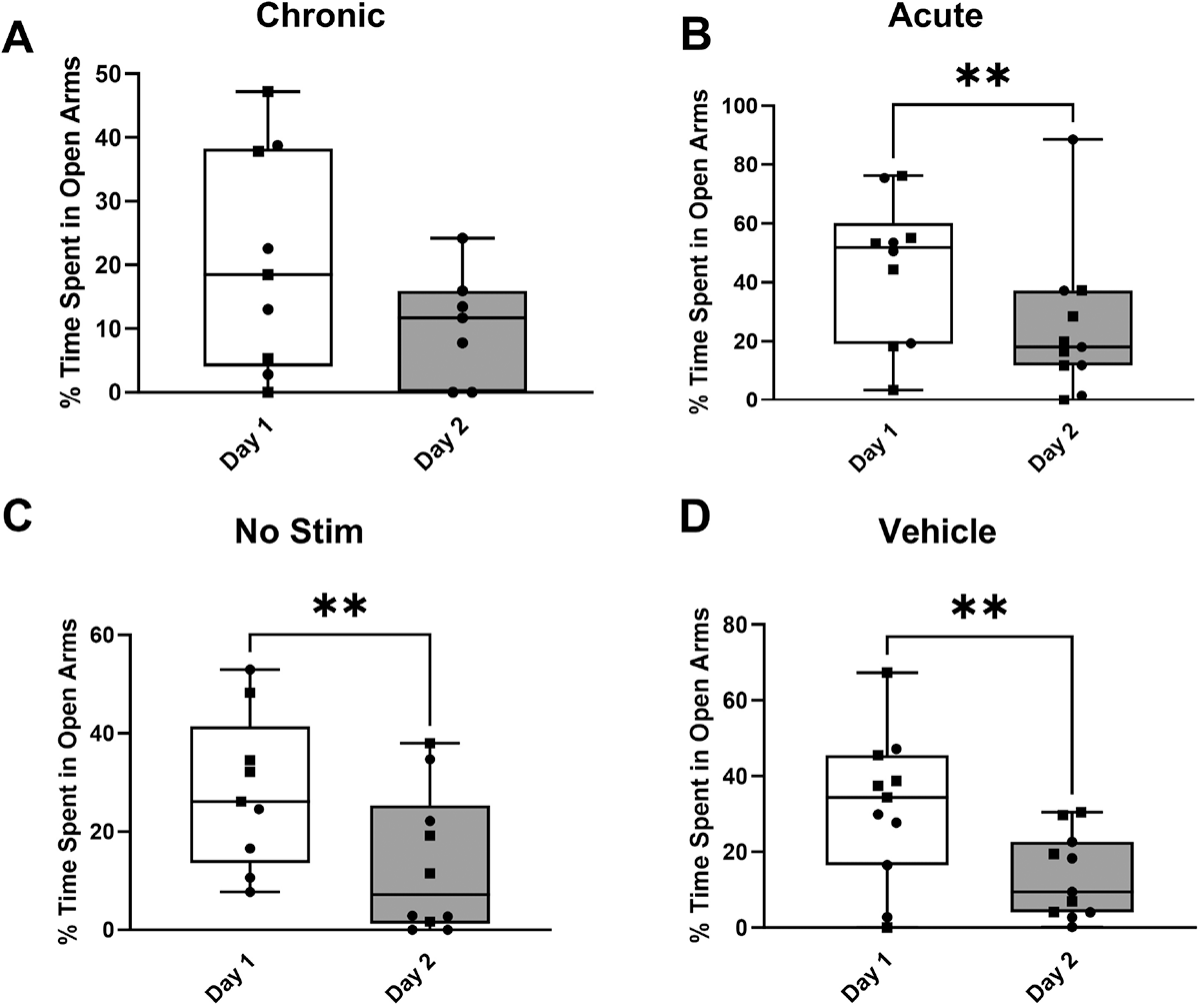
Percentage time spent in the open arms during second exposure to the elevated zero maze. Rats spent significantly less time (** *p* < 0.01) in the open arms during the second exposure to the elevated zero maze (day 22), suggesting sensitization to the elevated zero maze after a single exposure in all groups except the chronically stimulated group. (A) Chronically stimulated rats (W = −14.0, *p* = 0.297) (n = 10 on day 21, n = 8 on day 22). (B) Acutely stimulated rats (W = −55.0, *p* = 0.002) (n = 10 on day 21, n = 11 on day 22). (C) Lesioned rats that were not stimulated (W = −43.0, *p* = 0.008) (n = 9 on day 2, n = 10 on day 22). (D) Non-stimulated vehicle controls (W = −60.0, *p* = 0.005) (n = 11 on day 21, n = 11 on day 22). Data are presented using box and whisker plots. The median is denoted by the line inside the box, and the box edges signify the lower (25%) and upper (75%) quartiles. Squares represent female rats and circles represent male rats. A *p* value of 0.05 was considered significant using the Mann-Whitney test.

**Fig. 5.**
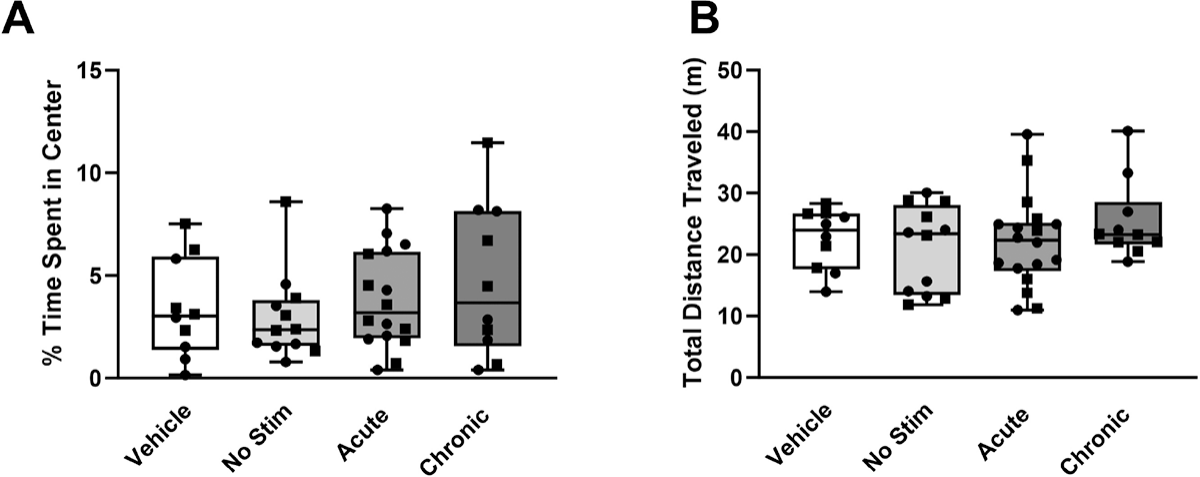
Risk-avoidant behavior during the first 5 minutes in the open field test. Risk-avoidant behavior during the open field test for each of the experimental groups: chronically stimulated (n = 11), acutely stimulated (n = 16), lesioned but not stimulated (n = 12), and non-stimulated vehicle controls (n = 10). Stimulation did not evoke significant changes in (A) percentage of time spent in the center of the field (H = 2.38, *p* = 0.498), or (B) total distance traveled (*p* = 1.47, H = 0.689) compared with rats that did not receive subthalamic nucleus deep brain stimulation. Data are presented using box and whisker plots, the median is denoted by the line inside the box, and the box edges signify the lower (25%) and upper (75%) quartiles. Squares represent female rats and circles represent male rats. Significant changes in risk-avoidant behaviors compared with rats who were not stimulated. A *p* value of 0.05 was considered significant using the Kruskal-Wallis test.

Notably, significant decreases in the median percentage of time spent in the open arms were observed for rats in the acute stimulation group (W = −55.0, *p* = 0.002) from 51.83% to 18.00%, the no stimulation group (W = −43.0, *p* = 0.008) from 26.13% to 7.20%, and the vehicle group (W = −60.0, *p* = 0.005) from 34.37% to 9.40% (Fig. 4B–D, respectively) after 1 day of prior exposure to the maze. In the open field test, there were no significant differences in the percentage of time spent in the center of the open field (Fig. 5A, H = 2.38, *p* = 0.498) or total distance traveled (Fig. 5B, H = 1.47, *p* = 0.689) between the experimental groups. The results for both the elevated zero maze and the open field test remained consistent when accounting for the sex of the animal (Supplementary Figs. 1–6).

### 3.2 Anxiogenic Control

**Fig. 6.**
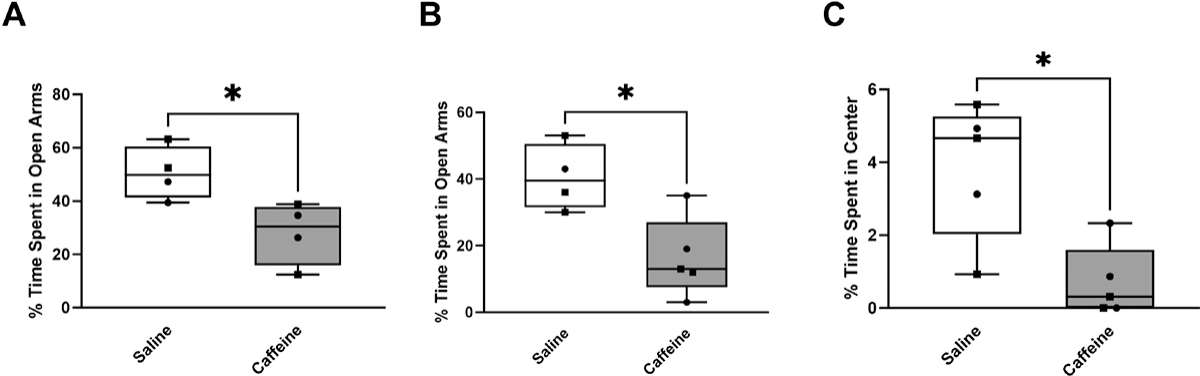
Validation of behavioral paradigm using an anxiogenic control. Percentage of time spent in the open arms of the elevated zero maze (A) during the first exposure (day 21) for the saline group (n = 4) and caffeine group (n = 5). (B) during second exposure (day 22) for the saline (n = 6) and caffeine (n = 5) groups. (C) Percentage of time spent in the center of the open field test for both the saline (n = 5) and caffeine (n = 5) groups. Rats that received caffeine (anxiogenic control) spent significantly less time (U = 0, *p* = 0.029) in the open arms compared with rats that received saline. This behavior remained consistent during the second exposure to the elevated zero maze (U = 2, *p* = 0.017). An increase in risk-avoidant behaviors was also observed in the open field test (U = 1, *p* = 0.016). Caffeine administration evoked anxiogenic behaviors in both the elevated zero maze and open field test. Data are presented using box and whisker plots. The median is denoted by the line inside the box, and the box edges signify the lower (25%) and upper (75%) quartiles. Squares represent female rats and circles represent male rats. A *p* value of 0.05 was considered significant using the Mann-Whitney test (* *p* < 0.05).

A separate cohort of rats was evaluated in the elevated zero maze as positive controls to a known anxiogenic agent, caffeine. Rats in the elevated zero maze that received a caffeine injection spent a median time of 30.47% in the open arms, which was significantly less (U = 0, *p* = 0.029) than those who received a saline injection, which spent a median time of 49.80% in the open arms (Fig. 6A). This behavior remained consistent even after 1 day of prior exposure to the maze (Fig. 6B). On the second day in the maze, the group treated with caffeine spent a median time of 13.13% in the open arms, which was significantly less (U = 2, *p* = 0.017) than the median time of 43.32% in the open arms spent by the group treated with saline. Rats evaluated in the open field test that received caffeine spent a median time of 0.31% in the center of the open field (Fig. 6C), in contrast to a median time of 4.66% spent in the center of the open field observed in the cohort who received saline (U = 1, *p* = 0.016).

### 3.3 Immunohistologic Analysis

**Fig. 7.**
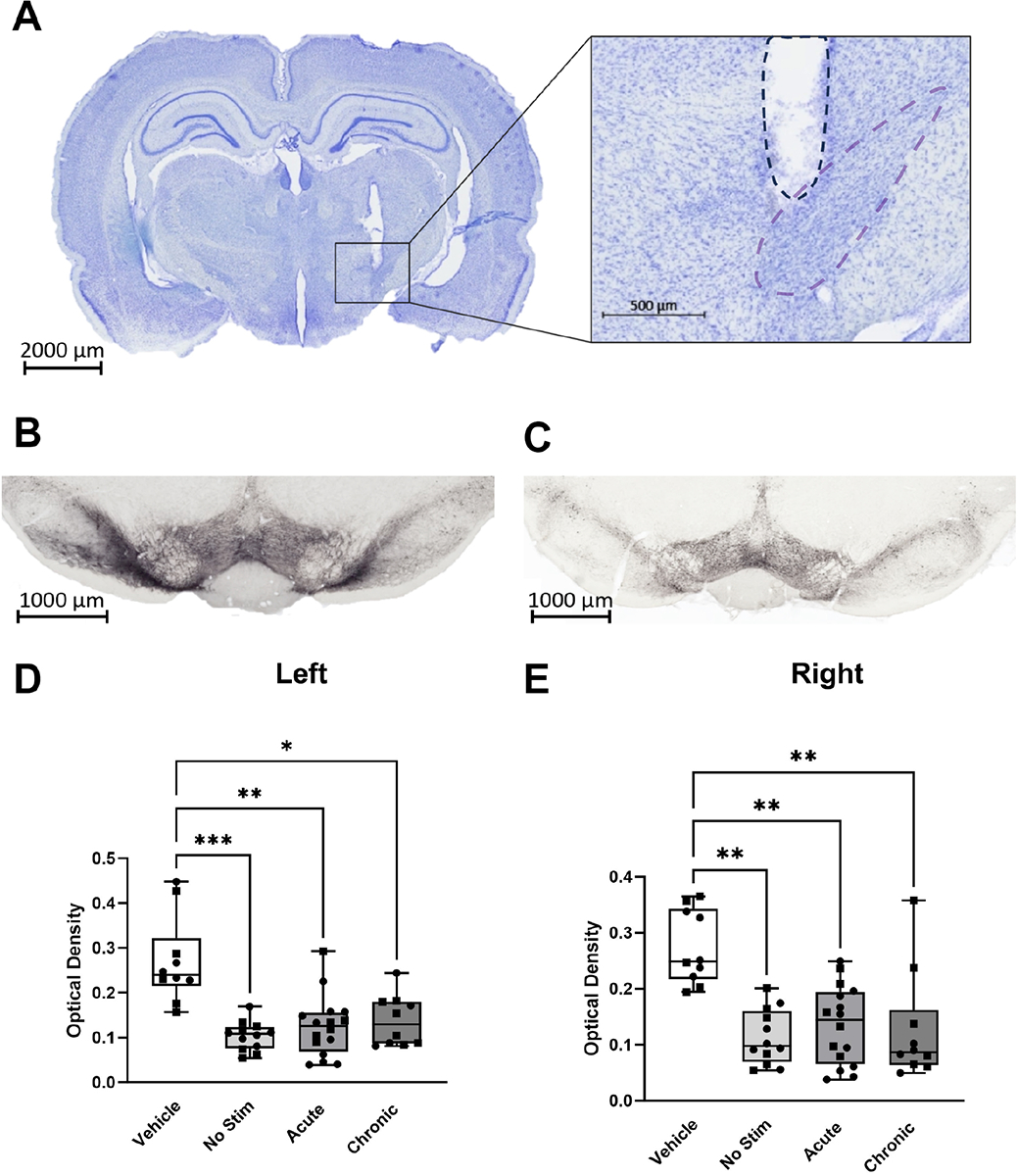
Histological analysis. Cresyl violet staining was used to confirm electrode location within the subthalamic nucleus (STN) and appropriate lesion level. (A) Typical brain slice staining showing electrode location denoted by the ensuing tissue lesion. The inset shows a magnified view of the electrode location (marked by the black dashed line) and the STN (marked by the purple dashed line) (ML 2.5, AP −3.6, DV −7.8) [39]. The slice shown was collected 3.72 mm posterior to the bregma [39]. Typical example of brain slices showing immunohistology of TH+ cells in the substantia nigra pars compacta in the (B) vehicle and (C) partial 6-OHDA-lesioned cohorts, respectively. Both images shown are located 5.88 mm posterior to the bregma [39]. (D) Optical density analysis of TH+ cells in each experimental group in the (D) left and (E) right hemispheres used to confirm the dopaminergic lesions. For the left hemisphere, analysis showed a decrease in the number of TH+ cells across all lesioned groups compared with the vehicle group (* *p* < 0.05, *** *p* < 0.001). Chronic compared with vehicle (*p* = 0.035), acute compared with vehicle (*p* = 0.0005), and no stimulation compared with vehicle (*p* = 0.0004). No statistically significant differences (chronic compared with acute *p* > 0.999, acute compared with no stim *p* > 0.999, chronic compared with no stim *p* > 0.999) were observed between the groups that received the lesion. For the right hemisphere, analysis showed a notable decrease in the number of TH+ cells across all lesioned groups when compared with the vehicle control group (** *p* < 0.01). Chronic compared with vehicle (*p* = 0.0025), acute compared with vehicle (*p* = 0.0018), and no stimulation compared with vehicle (*p* = 0.0014). No statistically significant differences (chronic compared with acute *p* > 0.999, acute compared with no stim *p* > 0.999, chronic compared with no stim *p* > 0.999) were observed between the lesioned groups. Data are presented using box and whisker plots. The median is denoted by the line inside the box, and the box edges signify the lower (25%) and upper (75%) quartiles. Squares represent female rats and circles represent male rats. A *p* value of 0.05 was considered significant using the Kruskal-Wallis test followed by Dunn’s multiple comparisons test. ML, Medial Lateral; AP, Anterior Posterior; DV, Dorsal Ventral.

We validated the accurate placement of electrodes within the STN using cresyl violet staining (n = 72) (Fig. 7A, Ref. [39]). Rats with electrodes placed outside of the target STN region (n = 7) were excluded from the study. We confirmed and quantified the extent of the dopaminergic lesion in the substantia nigra pars compacta through TH staining (Fig. 7B,C). Immunohistologic analysis showed a significant (*p* < 0.035) decrease in optical density across all lesioned groups when compared with the vehicle control group (Fig. 7D,E). Furthermore, no statistically significant differences (*p* > 0.999 for all comparisons) were observed between the groups that received the lesion (Fig. 7D,E).

## 4. Discussion

PD is primarily characterized by motor symptoms such as tremor, rigidity, and bradykinesia. However, it is also associated with a range of non-motor symptoms such as anxiety, which can manifest as quantifiable risk-avoidant behaviors. This study builds on previous work [32–34] to investigate the effect of STN DBS on risk-avoidant behaviors by using a pre-motor parkinsonian animal model that avoids behavioral confounds associated with impaired mobility. Maximum dopaminergic degeneration has been reported to take place at day 21 following 6-OHDA lesioning [4]. It is worth noting, however, that STN DBS was started on day 14 to ensure that the chronically-stimulated group received 1 week of stimulation prior to beginning behavioral assessments, in order to compare the effects of prolonged stimulation with the effects of acute stimulation. A significant decrease in dopaminergic neurons within the substantia nigra, as indicated by reduced optical density in the substantia nigra confirmed by TH staining (Fig. 7D), confirms that 6-OHDA induced an effective dopaminergic lesion.

The elevated zero maze test measures the balance between a rat’s innate aversion to open spaces and its motivation to explore novel environments. As such, rats that spend more time in the closed arms and avoid the open arms exhibit behaviors associated with anxiety and fear. Conversely, rats that spend more time in the open arms and make frequent entries into these areas are considered less anxious and more willing to explore open environments. We hypothesized that STN DBS would decrease risk-avoidant behaviors in a partial bilateral 6-OHDA lesioned animal model. However, the results of this study identified no significant changes in risk-avoidant behaviors between experimental groups that received STN DBS and those that did not receive stimulation (Fig. 2). The acute stimulation group showed a statistically significant increase in head dips relative to the other groups (Fig. 2C, *p* = 0.0169), which suggests a decrease in risk-avoidant behaviors [33,34]. Similarly, there were no differences between the stimulated and non-stimulated groups on the second exposure to the elevated zero maze (Fig. 3). The open field test was used to assess exploratory behavior. In general, anxious rats spend less time in the central, more exposed areas of the field, while total distance traveled is a measure of overall locomotor function. Similar to the results of the elevated zero maze, there were no differences between the stimulated and non-stimulated groups, suggesting that no motor deficits were induced by stimulation (Fig. 5). There were also no significant differences (*p* > 0.273) in the total distance traveled in either the open field (H = 1.47, *p* = 0.689) or elevated zero maze on either the first (day 21) or second (day 22) exposures to the open field test (H = 3.89, *p* = 0.273 and H = 2.36, *p* = 0.501, respectively), further suggesting that the stimulation did not induce motor impairments. This confirms that the results observed in this study were not caused by the animal’s inability to move freely throughout the maze. These results remained consistent when accounting for sex differences (Supplementary Figs. 1–6). A limitation of this study was the inability to obtain a baseline measurement in the same rats prior to lesioning or electrode implantation due to habituation to the environment. Additionally, the chronic stimulation protocol involved daily stimulation for 2 hours, and this may not have been sufficient to evoke behavioral changes. Rats demonstrated sensitization to the elevated zero maze after a single exposure in all groups except the chronically stimulated group. A reduction in time spent in the open arms of the elevated zero maze was statistically significant in the acute (W = −55.0, *p* = 0.002), unstimulated (W = −43.0, *p* = 0.008), and vehicle (W = −60, *p* = 0.005) groups. Only the chronically stimulated group (Fig. 4A) did not show a significant increase in risk-avoidant behaviors (W = −14.0, *p* = 0.297). Previous studies have shown decreased exploratory behaviors in rats after interacting with the elevated zero maze, which demonstrates sensitization to the maze [47–49]. This suggests that chronic stimulation may cause a decrease in risk-avoidant behaviors over time. However, additional research is needed to improve understanding of the long-term implications of chronic stimulation in animal models.

We administered caffeine, a well-known anxiogenic, as a positive control in a group of naïve rats to both validate the results observed and ensure the robustness of our experimental approach [44,45]. Our results confirmed the sensitivity of both the elevated zero maze and open field test in detecting changes in risk-avoidant behaviors among rats that received either caffeine or saline. However, this cohort did not undergo surgery nor tethering. Furthermore, a different caffeine dose was reported depending on the behavioral test administered (100 mg/kg for the elevated zero maze and 50 mg/kg for the open field test). The difference in caffeine dose was due to an error with the video capturing system during the open field test for rats that received 100 mg/kg. That is, we collected behavioral data for the elevated zero maze at both 50 and 100 mg/kg but were unable to analyze the behavioral videos for the rats that received 100 mg/kg. To minimize animal usage and considering that the 50 mg/kg dose exhibited efficacy comparable with that observed with 100 mg/kg, a prior trial using 50 mg/kg was used for the analysis.

The role of the STN in risk-avoidant and other non-motor behaviors is an ongoing area of research. For example, recent studies have explored the role of the STN in aversive learning [50], which has many similarities to risk-avoidant behaviors, and demonstrated that selective stimulation of the STN contributes to aversion and conditioned avoidance in a rodent model. This supports a key role for the STN in other non-motor activities such as aversive learning and risk-avoidant behaviors. While our results did not show significant changes in risk-avoidant behaviors following 6-OHDA lesion, other studies have reported reduction in risk-avoidant behaviors following STN stimulation [33,34]. However, there are some notable differences that must be highlighted. While all studies used 6-OHDA lesioned rats, all lesions were different. Faggiani *et al*. [33] and Badstuebner *et al*. [34] injected 6-OHDA into the medial forebrain bundle (MFB) rather than the striatum, suggesting that MFB may be more effective in inducing risk-avoidant symptoms. Additionally, Faggiani *et al*. [33] and Badstuebner *et al*. [34] noted a decrease in motor activity post-lesion, a factor that may have confounded the results of the subsequent behavioral evaluations. Our study did not have a similar decrease in motor activity due to the model selected, thereby underscoring the complexity of interpreting behavioral outcomes in the context of motor changes induced by lesions. Grembecka *et al*. [51] also observed a decrease in risk-avoidant behaviors in addition to an improvement in food-related motivation following STN stimulation in rats that had received lesioning 7 days prior to stimulation. The method of lesioning may be responsible for the differences in outcome observed in both studies. Additional differences with these studies such as laterality of the stimulation (unilateral or bilateral) and electrode configuration (unipolar or bipolar) may also contribute to the outcome differences between our results and those referenced above. In the study presented here, rats had a stimulating electrode surgically implanted into the STN and were tethered to a stimulator placed above the elevated zero maze or open field test. A comparison of all groups (Figs. 4D,5,6) suggested that the combination of surgery and tethering can cause an increase in risk-avoidant behaviors in the elevated zero maze but not in the open field test. Thus, it is possible that the discrepancy between the behavioral differences observed in this study and previous studies may be attributed to other factors such as implantation surgery, stimulation paradigm, animal handling, habituation, tethering, and more. An additional factor potentially impacting the results of this study is the use of a bilateral 6-OHDA lesion with unilateral stimulation. A unilateral stimulating electrode was selected due to the limitations of our stimulation devices that prevented simultaneous chronic stimulation of multiple rats. This could have created a lateral effect that impacted behavior in response to stimulation. Additionally, all electrodes were implanted on the right hemisphere, which, while improving technical consistency, introduces a bias in the data that should be noted when interpreting these results. Moreover, STN DBS for PD is typically reserved for the later stages of disease progression. However, this study used DBS in a partially lesioned premotor PD model. This is both a feature and a limitation, as a partial lesion was selected to minimize motor impairments but fails to capture the true nature of PD. Future work should investigate the effects of STN DBS on a fully lesioned model while considering other methods to address motor deficits. Additionally, the effect of stimulating lesioned versus non-lesioned rats should be investigated, as this study focused on lesioned rats.

## 5. Conclusions

Our results suggest that unilateral STN DBS does not induce substantial changes in risk-avoidant behaviors in a partially lesioned premotor 6-OHDA model of PD. While this could be interpreted as STN DBS having a less critical role on changes in anxiety following STN DBS, further work is needed to further understand the impact of stimulation. Our results showed that chronic stimulation did prevent sensitization to the elevated zero maze, suggesting a possible effect of long-term stimulation on the progression of risk-avoidant behaviors. Future work should explore additional anxiety-related behaviors beyond risk-avoidance, which may better capture the mechanisms of anxiety, and should evaluate other animal models of anxiety that may provide better insight into the cause of anxiety following STN DBS. Finally, future work should consider the duration of stimulation applied prior to behavioral assessments, as there may be neuroplastic changes arising from longer periods of stimulation that are not captured in the stimulation duration used in this study.

## Supplementary Material

Supplementary Material

## Data Availability

The datasets used and/or analyzed during the current study are available from the corresponding author on reasonable request.
