# Supplementary material for: Effects of Unilateral High Frequency Stimulation of the Subthalamic Nucleus on Risk-avoidant Behavior in a Partial 6-hydroxydopamine Model of Parkinson’s Disease
Source: J Integr Neurosci. Author manuscript; Available in PMC 2024 Jul 11. (PMC11238881; doi:10.31083/j.jin2304084)
Supplement: Supplementary Material [file NIHMS2007349-supplement-Supplementary_Material.docx]

# Supplementary Material


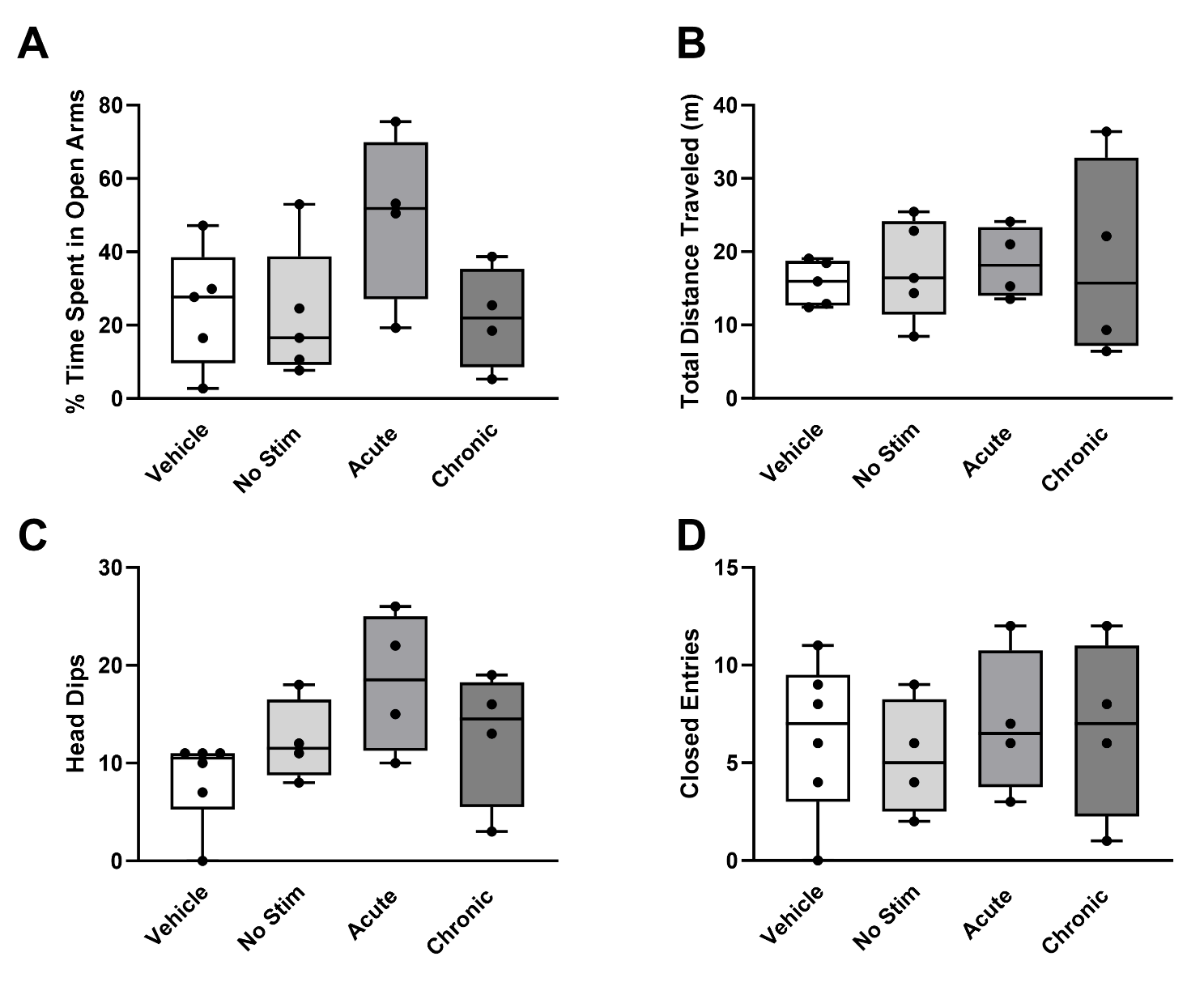


**Supplementary Fig. 1. Risk-avoidant behavior in male rats during their first exposure (day 21) to the elevated zero maze**. Risk-avoidant behavior for male rats in each experimental group during their fist exposure to the elevated zero maze: chronically stimulated (n = 4), acutely stimulated (n = 4), lesioned but not stimulated (n = 5), and non-stimulated vehicle controls (n = 5). Stimulation did not evoke significant changes in (A) Percentage of time spent in open arms, (B) Total distanced traveled, (C) Number of head dips, or (D) Number of entries into the closed arms compared to male rats that were not stimulated. Data are presented using box and whisker plots. The median is denoted by the line inside the box, and the box edges signify the lower (25%) and upper (75%) quartile.


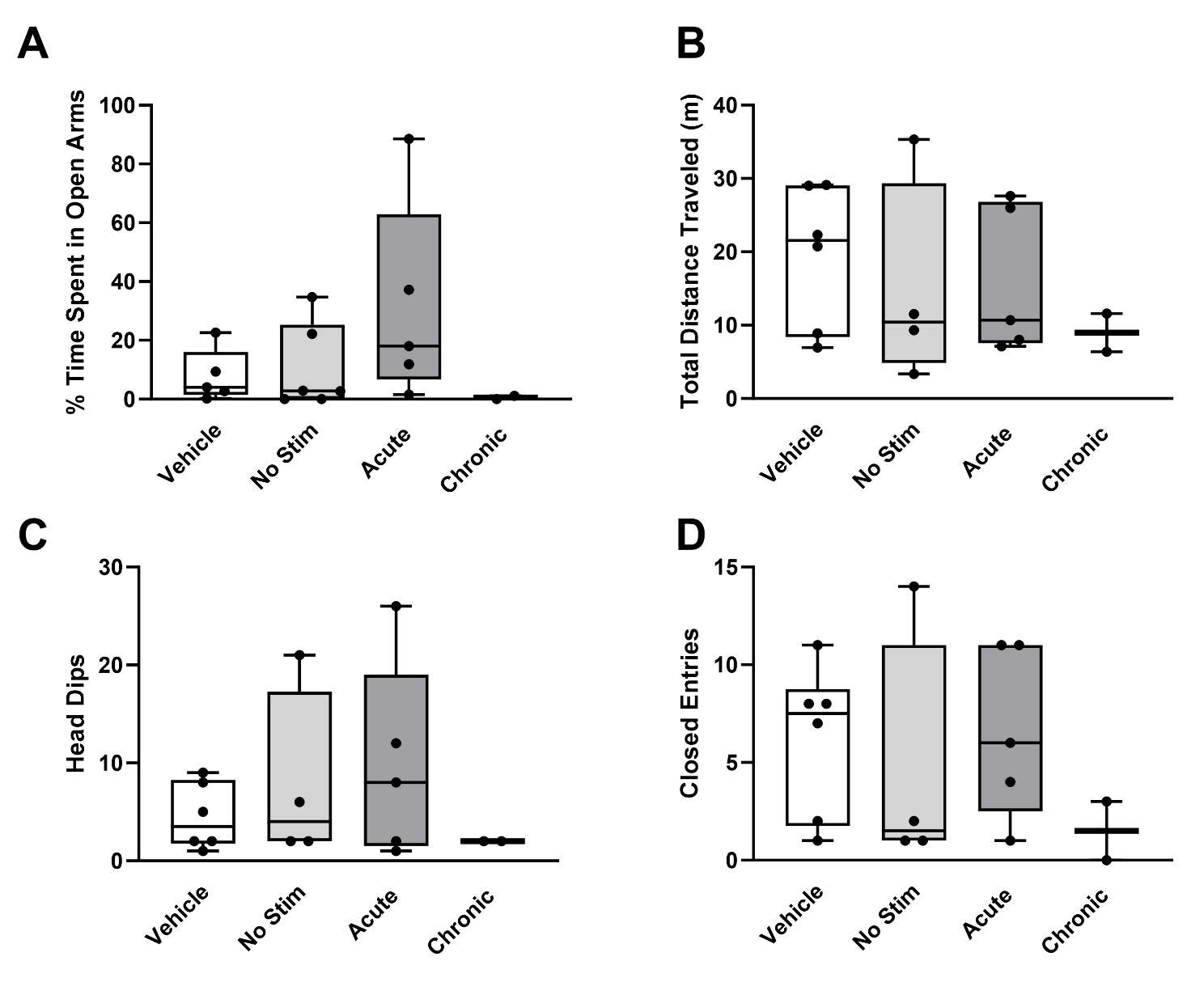


**Supplementary Fig. 2. Risk-avoidant behavior in male rats during their second exposure (day 22) to the elevated zero maze**. Risk-avoidant behavior for male rats in each experimental group during their second exposure to the elevated zero maze: chronically stimulated (n = 2), acutely stimulated (n = 5), lesioned but not stimulated (n = 4), and non-stimulated vehicle controls (n = 6). Stimulation did not evoke significant changes in (A) Percentage of time spent in open arms, (B) Total distanced traveled, (C) Number of head dips, or (D) Number of entries into the closed arms compared to male rats that were not stimulated. Data are presented using box and whisker plots. The median is denoted by the line inside the box, and the box edges signify the lower (25%) and upper (75%) quartile.


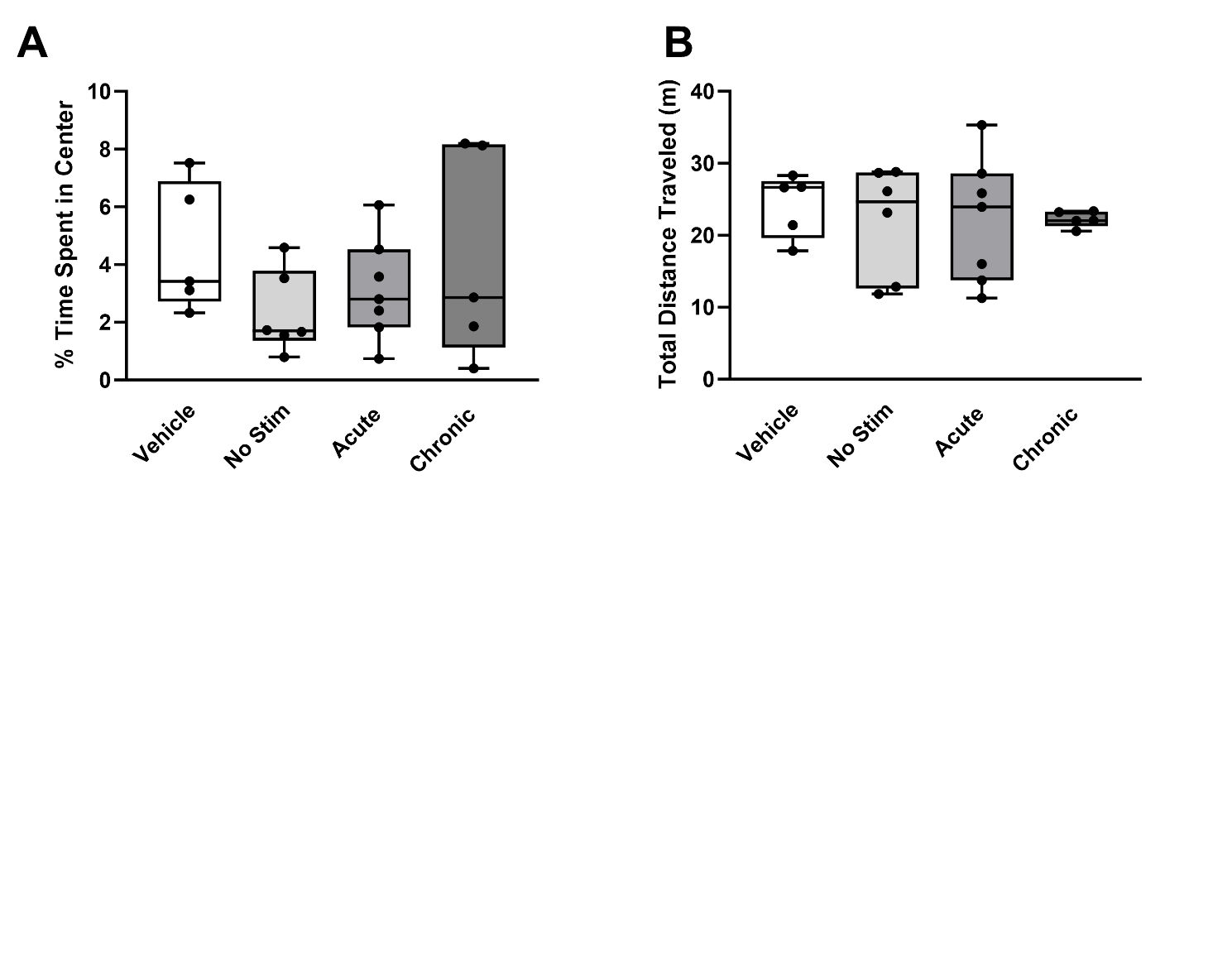


**Supplementary Fig. 3. Risk-avoidant behavior in male rats during the open field test.** Risk-avoidant behavior for male rats in each of the experimental groups during their exposure to the open field test: chronically stimulated (n = 5), acutely stimulated (n = 7), lesioned but not stimulated (n = 6), and non-stimulated vehicle controls (n = 5). Stimulation did not evoke significant changes in (A) Percentage of time spent in the center of the field or (B) Total distance traveled compared to male rats that were not stimulated. Data are presented using box and whisker plots. The median is denoted by the line inside the box, and the box edges signify the lower (25%) and upper (75%) quartile.


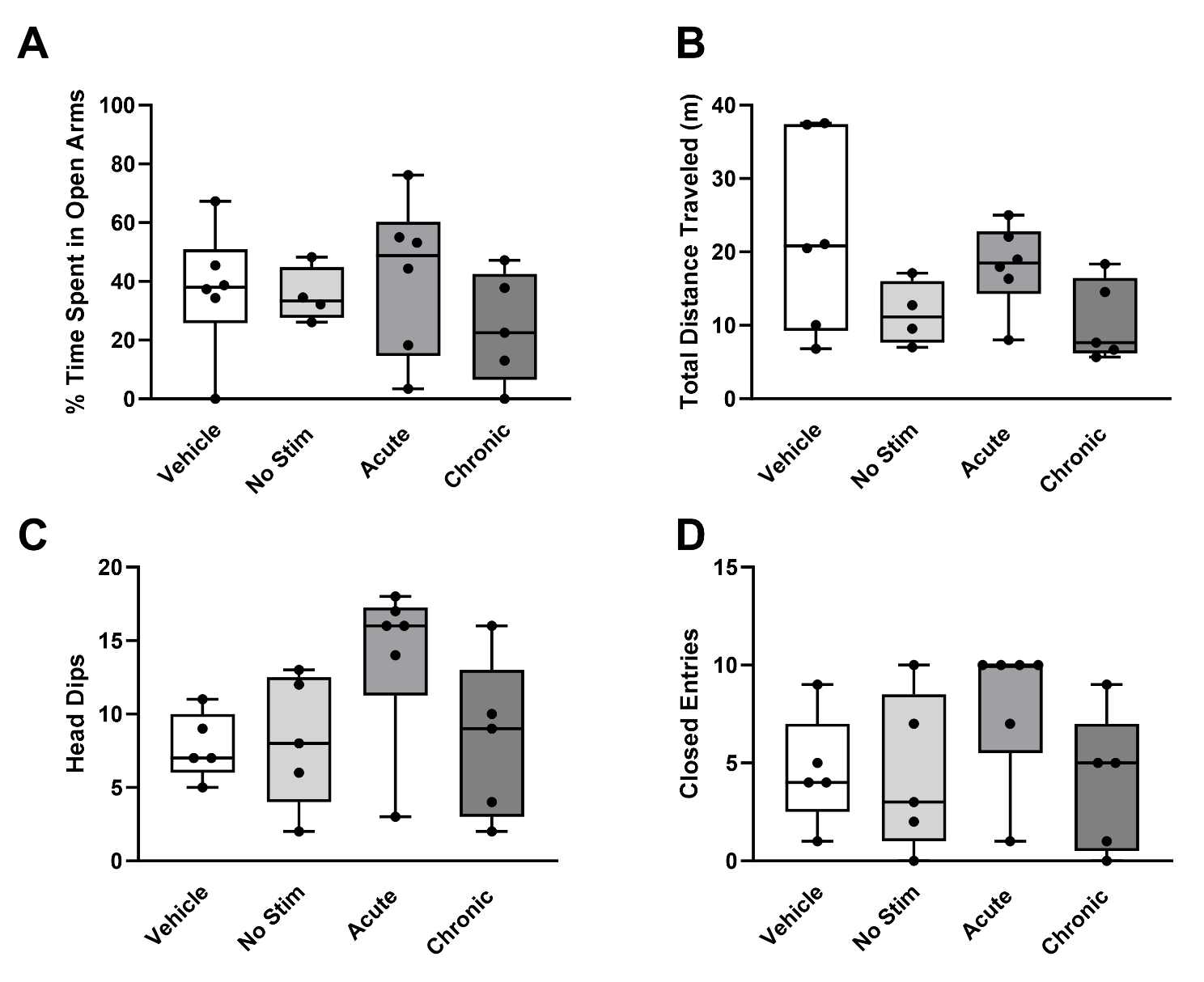


**Supplementary Fig. 4. Risk-avoidant behavior in female rats during their first exposure (day 21) to the elevated zero maze**. Risk-avoidant behavior in female rats in each of the experimental groups during their exposure to the elevated zero maze: chronically stimulated (n = 5), acutely stimulated (n = 6), lesioned but not stimulated (n = 4), and non-stimulated vehicle controls (n = 5). Stimulation did not evoke significant changes in (A) Percentage of time spent in open arms, (B) Total distanced traveled, (C) Number of head dips, and (D) Number of entries into the closed arms compared to female rats that were not stimulated. Data are presented using box and whisker plots. The median is denoted by the line inside the box, and the box edges signify the lower (25%) and upper (75%) quartile.


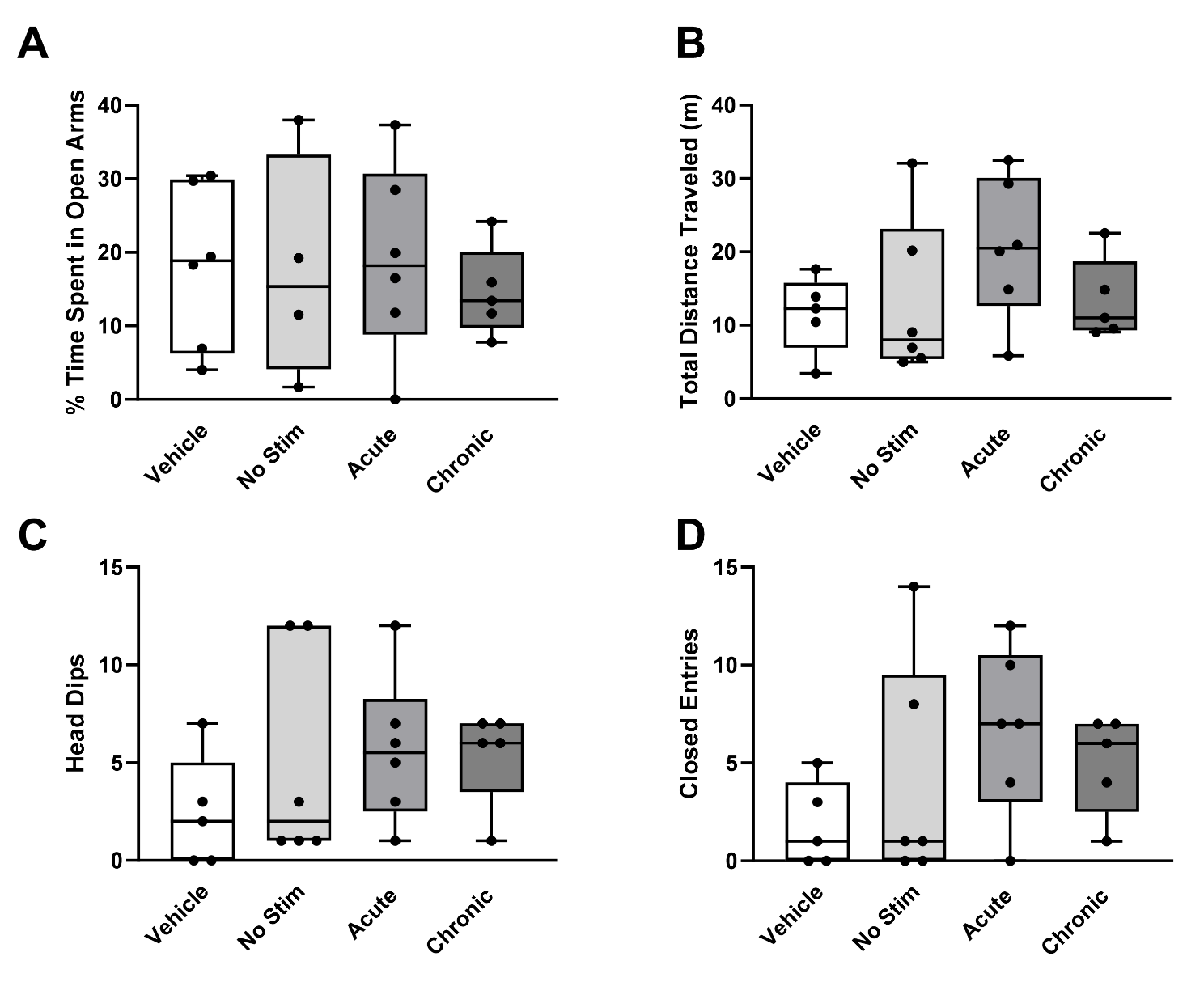


**Supplementary Fig. 5. Risk-avoidant behavior in female rats during their second day of exposure (day 22) to the elevated zero maze.** Risk-avoidant behavior for female rats in each of the experimental groups during their exposure to the elevated zero maze: chronically stimulated (n = 5), acutely stimulated (n = 6), lesioned but not stimulated (n = 4), and non-stimulated vehicle controls (n = 5). Stimulation did not evoke significant changes in (A) Percentage of time spent in open arms, (B) Total distanced traveled, (C) Number of head dips, or (D) Number of entries into the closed arms compared to female rats that were not stimulated. Data are presented using box and whisker plots. The median is denoted by the line inside the box, and the box edges signify the lower (25%) and upper (75%) quartile. Squares represent female rats and circles represent male rats.


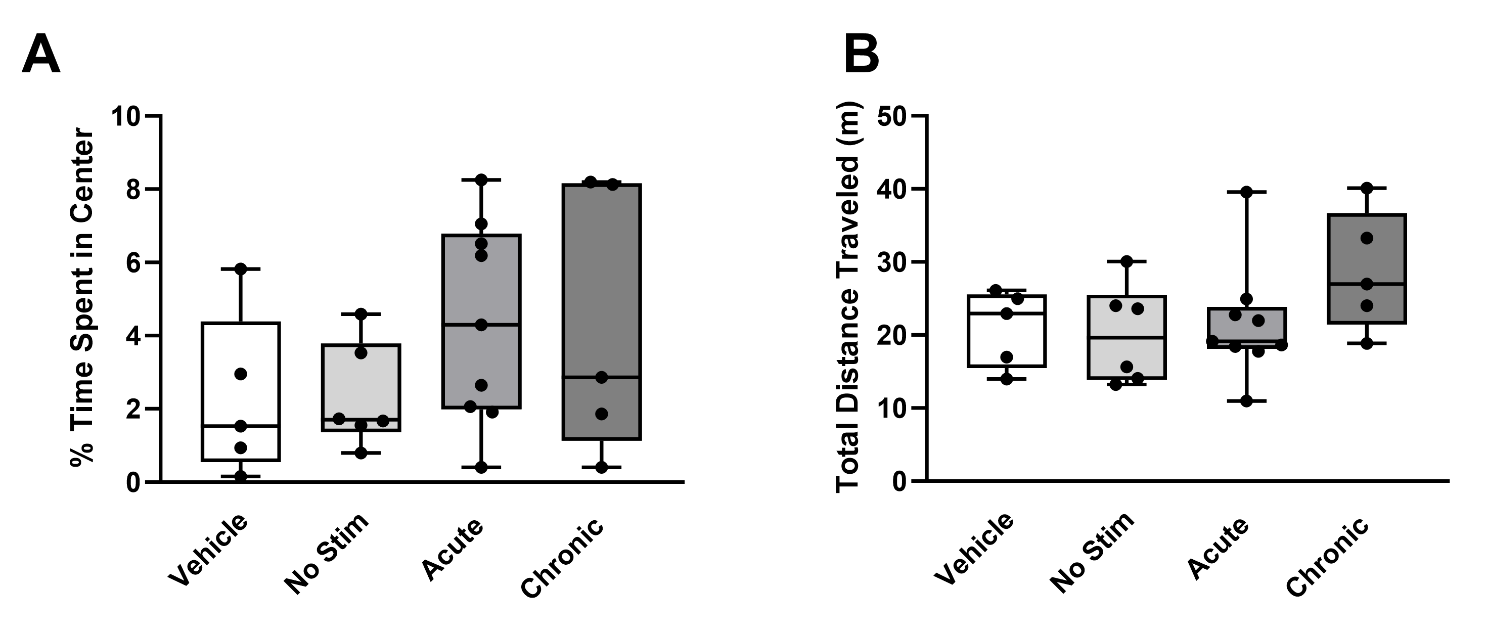


**Supplementary Fig. 6. Risk-avoidant behavior in female rats during their exposure to the open field test**. Risk-avoidant behavior for female rats in each of the experimental groups during their exposure to the open field test: chronically stimulated (n = 5), acutely stimulated (n = 9), lesioned but not stimulated (n = 6), and non-stimulated vehicle controls (n = 5). Stimulation did not evoke significant changes in (A) Percentage of time spent in the center of the field or (B) Total distance traveled compared to female rats that were not stimulated. Data are presented using box and whisker plots. The median is denoted by the line inside the box, and the box edges signify the lower (25%) and upper (75%) quartile.
